# Immunization of Cattle With Recombinant Structural Ectodomains I and II of *Babesia bovis* Apical Membrane Antigen 1 [BbAMA-1(I/II)] Induces Strong Th1 Immune Response

**DOI:** 10.3389/fvets.2022.917389

**Published:** 2022-06-23

**Authors:** Amarin Rittipornlertrak, Boondarika Nambooppha, Anucha Muenthaisong, Nisachon Apinda, Pongpisid Koonyosying, Wanwisa Srisawat, Paweena Chomjit, Kanokwan Sangkakam, Veerasak Punyapornwithaya, Saruda Tiwananthagorn, Naoaki Yokoyama, Nattawooti Sthitmatee

**Affiliations:** ^1^Laboratory of Veterinary Vaccine and Biological Products, Faculty of Veterinary Medicine, Chiang Mai University, Chiang Mai, Thailand; ^2^Ruminant Clinic, Department of Food Animal Clinics, Faculty of Veterinary Medicine, Chiang Mai University, Chiang Mai, Thailand; ^3^Department of Veterinary Bioscience and Veterinary Public Health, Faculty of Veterinary Medicine, Chiang Mai University, Chiang Mai, Thailand; ^4^National Research Center for Protozoan Diseases, Obihiro University of Agriculture and Veterinary Medicine, Obihiro, Japan; ^5^Excellent Center in Veterinary Bioscience, Chiang Mai University, Chiang Mai, Thailand

**Keywords:** *Babesia bovis*, AMA-1, recombinant protein, Th1 immune response, cattle, vaccine

## Abstract

Both strong innate and adaptive immune responses are an important component of protection against intraerythrocytic protozoan parasites. Resistance to bovine babesiosis is associated with interferon (IFN)-γ mediated responses. CD4^+^ T cells and macrophages have been identified as major effector cells mediating the clearance of pathogens. Previously, the apical membrane antigen 1 (AMA-1) was found to significantly induce the immune response inhibiting *B. bovis* merozoite growth and invasion. However, a detailed characterization of both humoral and cellular immune responses against the structure of *B. bovis* AMA-1 (BbAMA-1) has not yet been established. Herein, the present study aimed to express the recombinant BbAMA-1 domain I+II protein [rBbAMA-1(I/II)], which is the most predominant immune response region, and to characterize its immune response. As a result, cattle vaccinated with BbAMA-1(I/II) significantly developed high titters of total immunoglobulin (Ig) G antibodies and a high ratio of IgG2/IgG1 when compared to control groups. Interestingly, the BbAMA-1(I/II)-based formulations produced in our study could elicit CD4^+^ T cells and CD8^+^ T cells producing IFN-γ and tumor necrosis factor (TNF)-α. Collectively, the results indicate that immunization of cattle with BbAMA-1(I/II) could induce strong Th1 cell responses. In support of this, we observed the up-regulation of Th1 cytokine mRNA transcripts, including *IFN-*γ, *TNF-*α, Interleukin *(IL)-2* and *IL-12*, in contrast to down regulation of *IL-4, IL-6* and *IL-10*, which would be indicative of a Th2 cytokine response. Moreover, the up-regulation of inducible nitric oxide synthase (*iNOS*) was observed. In conclusion, this is the first report on the in-depth immunological characterization of the response to BbAMA-1. According to our results, BbAMA-1 is recognized as a potential candidate vaccine against *B. bovis* infection. As evidenced by the Th1 cell response, it could potentially provide protective immunity. However, further challenge-exposure with virulent *B. bovis* strain in immunized cattle would be needed to determine its protective efficacy.

## Introduction

Most apicomplexans are obligate intracellular parasites. *Babesia*, an apicomplexan parasite, is a tick-transmitted hemoprotozooan. The most economically relevant bovine babesias are *Babesia bovis* (*B. bovis*) and *Babesia bigemina* (*B. bigemina*). Presently, a large number of cattle are at risk of exposure to bovine babesiosis. Bovine babesiosis is recognized as a global disease that has been the cause of significant economic losses to the livestock industry (1). Climate change is one of the factors that can influence the control of bovine babesiosis (2). Pathologically, a cyclical asexual replication of *Babesia* in red blood cells (RBC) that is associated with an excessive immune response has been known to lead to the development of several clinical manifestations. In general, bovine babesiosis can be treated with chemotherapy (1). However, the emergence of drug resistance in *B. bovis* has been recently documented (3). Although live attenuated vaccines are available in some endemic countries, the applications for wide use of these vaccines are limited for a number of reasons. At present, there are no safe and effective vaccines that protect cattle against the virulent pathogens (4). Thus, novel vaccines are increasingly desirable and urgently needed to control this disease (2).

The host-parasite interaction is of major importance for parasite survival. Without host cells, which supply environment and nutrition, the parasitic protozoa cannot grow and survive. The formation of a moving junction (MJ) between the parasite and host cell membranes is an important conserved mechanism that facilitates the parasitic invasion into host cells during the asexual growth cycle of apicomplexan parasites (5). The apical membrane antigen 1 (AMA-1) has been well characterized and reported to be involved in MJ formation in coordination with the rhoptry neck 2 (RON2) protein in *Toxoplasma* and *Plasmodium* (6, 7). It is likely directly responsible for reorientation and initiates the junctional contact. AMA-1, a microneme protein (MIC), is a type I integral membrane protein that is composed of three distinct structures (8). This protein is not only well conserved among *Plasmodium* species (9) but also other *Apicomplexa* species including *Toxoplasma* (10), *Neospora* (11) and *Babesia* (12–14). Unfortunately, little is known about the biological function of AMA-1. Several lines of evidence have demonstrated that AMA-1 plays an essential role in the invasion process (6, 7, 10, 15) and is secreted onto the surface at or around the time of merozoite egression (16). Attempts to inactivate *ama-1* of *Plasmodium falciparum* (*P. falciparum*) (17) and *Toxoplasma gondii* (*T. gondii*) (18) have strongly supported the contention that AMA-1 plays a central role in merozoite invasion. Moreover, it either directly or indirectly plays a role in the resealing of the red blood cells at the posterior end of the invasion event, which would indicate that this protein may be essential to the survival of the parasite. However, it has been argued that AMA-1 plays a role in host cell attachment rather than MJ formation as a way of facilitating the invasion (19). Therefore, the importance of this protein continues to be debated.

Interestingly, AMA-1 is not only capable of involvement in parasitic invasion, it is also potentially an immunogen. Immunological studies in animal models have revealed that immunization with AMA-1 was able to induce significant protection against homologous but not heterologous malaria parasites (20–22). Accordingly, the molecules that are involved in immune system recognition and the steps of invasion are of great interest for the development of prophylaxis. For this reason, AMA-1 is a candidate antigen of significant interest for vaccine development against apicomplexan parasite infection. Given its important role and the immune response data, AMA-1 has been extensively studied as a potential long-standing malaria vaccine candidate (23). Genetically, a high degree of sequence polymorphisms was observed in domain I of the extracellular domain of *Plasmodium ama-1*. It has been suggested that the presence of the polymorphism is presumably due to host immune pressure (24, 25). The crystal structure reveals AMA-1 has a hydrophobic cleft that runs across domains I and II (26). This hydrophobic cleft was thought to be an AMA-1 ligand-binding site and a major target of protective immunity (27). Interestingly, it has been previously identified as the major target of the invasion-inhibitory monoclonal antibody (25, 27–29) and the invasion-inhibitory peptide (30, 31). Therefore, it is likely that domains I and II are the predominant targets for inhibitory responses and immunogenicity (32–34).

In consideration of the fact that domains I and II of AMA-1 are of vital importance to the apicomplexan parasite, this protein is of great interest as a candidate for subunit vaccine development against bovine babesiosis. Among *B. bovis* strain, *ama-1* has been highly conserved. Importantly, a strong negative or purifying selection across the whole of the gene was established, especially in domain I, indicating strong functional constraints on this gene (35). Interestingly, *B. bovis* AMA-1 (BbAMA-1) is recognized by the antibodies against the epitopes that are mainly situated within domain I (12, 36) and domain II (37). These antibodies could inhibit the *in vitro* growth and invasion of erythrocytes by *B. bovis* merozoites. Collectively, the results indicate that BbAMA-1 domains I and II are potential targets in the development of a promising bovine babesiosis vaccine. To the best of our knowledge, detailed characterizations of both humoral and cellular immune responses against the structure of BbAMA-1 have not yet been established. To gain a more comprehensive understanding of the immune response elicited by BbAMA-1, we have herein expressed the structure of domains I and II of BbAMA-1 (Ser^40^-Glu^438^) and characterized their immunological functions.

## Materials and Methods

### Animals

Twenty healthy 10–16-month-old Holstein Friesian dairy cows were included in this study. Dairy cattle were kept in a free-stall barn. All cattle were acquired from farm members under the Mae Wang Dairy Cooperative, Mae Wang District, Chiang Mai Province, Thailand. Each cow was screened for *B. bovis* infection by PCR, as has been previously described (35), and then subjected to the immunofluorescence antibody test (IFAT) (38).

### Recombinant BbAMA-1 Domain I+II Protein [rBbAMA-1(I/II)] Protein Expression and Purification

The sequence of ectodomains I and II *B. bovis ama-1* encoding Ser^40^-Glu^438^ was selected from GenBank accession number KY575957. Codons were optimized, synthesized (Genscript, Piscataway, NJ, United States) and inserted into pQE-32 vector (Qiagen GmbH, Hilden, Germany) with N-terminal *Sac*I and C-terminal *Hind*III enzyme sites. Accordingly, pQE-32/BbAMA-1(I/II) plasmid was transformed into *Escherichia coli* (*E. coli*) M15 strain using the heat shock method at 42°C for 45 s and then spread on Luria-Bertani (LB; Difco™, Sparks, MD, United States) agar containing 100 μg/ml ampicillin and 25 μg/ml kanamycin and incubated at 37°C for 16 h. A positive single colony detected by pQE specific vector primer (Primer-Type III/IV-pQE forward: CGGATAACAATTTCACACAG; Primer-pQE Reverse: GTTCTGAGGTCATTACTGG) was inoculated in LB broth (Difco™) containing 100 μg/ml ampicillin and 25 μg/ml kanamycin. It was then incubated in a shaking incubator at 250 rpm at a temperature of 37°C for 16 h for the purpose of starter culture preparation. One-liter of LB broth medium (LB broth, 100 μg/ml ampicillin and 25 μg/ml kanamycin) was inoculated in a ratio of 1:50 with the starter culture and continuously grown in the shaking incubator under the same growth conditions until an OD_600_ of 0.6 was reached. The recombinant protein expression was subsequently induced by the addition of isopropyl-β-D-thiogalactopyranoside (IPTG; Amresco, Solon, OH, United States) to a final concentration of 0.5 mM, while the culture was incubated for an additional 12 h. Finally, cells were harvested by centrifugation at 4,000 × g for 20 min at 4°C and kept at −20°C for purification.

The cell pellets were lysed in lysis buffer (Denaturing conditions; 100 mM NaH_2_PO_4_, 10 mM Tris-HCl, 8 M urea; pH 8.0) with gentle shaking at 4°C for 1 h. The suspension was then centrifuged at 10,000 rpm at 4°C for 30 min. The supernatant was transferred to new tubes and maintained at −80°C. The purification process of the recombinant 6xHis-tagged proteins in this study was conducted by employing Ni-NTA affinity chromatography according to the manufacturer's instructions (Qiagen GmbH, Hilden, Germany). The purified recombinant protein was dialyzed by gradually decreasing the urea concentration. The rBbAMA-1(I/II) protein was concentrated using Amicon® centrifugation filters (30 kDa MWCO; Merck KGaA, Darmstadt, Germany) and concentration was then measured using the BCA protein assay kit (Pierce®, Rockford, IL, United States) according to the manufacturer's instructions.

### Identification and Verification of rBbAMA-1(I/II) by Sodium Dodecyl Sulfate Polyacrylamide Gel Electrophoresis (SDS-PAGE) and Western Blot Analysis

Either lysate or purified protein was separated by 12.5% SDS-PAGE gel in a mini-slab apparatus (Bio-Rad Laboratories, Hercules, CA, United States). The process was run with 100 V for 1 h. The SDS-PAGE slab gels were then stained with Coomassie blue R-250 (Sigma-Aldrich, St. Louis, MO, United States) for protein band detection. Moreover, the proteins obtained from the SDS-PAGE gel were electrically transferred onto a nitrocellulose membrane (Merck Millipore™, Merck KGaA, Darmstadt, DEU) at 15 V for 1 h. The membrane was then incubated with previously produced rabbit polyclonal anti-sBbAMA-1 serum (1:50 dilution) at 4°C overnight (36). Subsequently, the membrane was probed with horseradish peroxidase (HRP)-conjugated goat anti-bovine immunoglobulin (Ig) G antibody (1:2,000 dilution; KPL, Gaithersburg, MD, United States). The membrane was then incubated with gentle shaking at room temperature for 1 h and then washed three times with washing buffer. Finally, the reactions were visualized using a solution containing 3,3'-diaminobenzidine (DAB; Invitrogen, Carlsbad, CA, United States) and hydrogen peroxide (H_2_O_2_; Merck, Germany).

### Vaccination Program

Vaccine formulations were prepared by mixing different rBbAMA-1(I/II) concentrations (50 and 100 μg) with the Montanide™ ISA 206 VG adjuvant (1:1 V/V, SEPPIC, Paris, France) to a total volume of 1 ml/dose. The phosphate-buffered saline (PBS) formulated with only the adjuvant was used as a control. The vaccines were freshly prepared and stored at 4°C until being used. Cattle were divided into four groups based on the vaccine formulations (Table 1). All groups were intramuscularly immunized four times at 2-week intervals with the exception of the non-immunized group. Blood samples were collected before immunization and every 2 weeks for 10 weeks in order to determine the immunological responses. Adverse events, including pain, swelling at the injection site and behavioral changes, were monitored throughout the course of the experiment.

**Table 1:** Vaccine immunization in cattle.

**Group**	**Vaccine formulation**	**Animal/group**
1	100 μg of rBbAMA-1(I/II) + Montanide ISA 206 VG	5
2	50 μg of rBbAMA-1(I/II) + Montanide ISA 206 VG	5
3	Montanide ISA 206 VG + PBS	5
4	Non-immunization	5
Total		20

### Determination of Humoral Immune Response Using Indirect Enzyme-Linked Immunosorbent Assay (ELISA) Procedure

The immunoplate (Nunc-immuno^TM^ plate, Denmark) was coated with 100 μg of rBbAMA-1(I/II) protein in a coating buffer (0.05 M carbonate bicarbonate buffer, pH 9.6) and then incubated overnight at 4°C. Unbound antigens were washed three times with a washing buffer [PBS (pH 7.2) containing 0.05% Tween-20; PBST]. Non-specific bindings were blocked with a blocking buffer (1% skim milk in PBS) and the plate was incubated at room temperature for 1 h. After thrice washing, cattle serum samples at a dilution of 1:50 in the blocking buffer were added to the wells in duplicate and they were then incubated at room temperature for 1 h. After being washed thrice with the washing buffer, HRP-conjugated goat anti-bovine IgG antibody (KPL, Gaithersburg, MD, United States) was used as the secondary antibody at a dilution of 1:2000 in the blocking buffer and incubated at room temperature for 1 h. After being washed three times, 3,3′,5,5′-tetramethylbenzidine (TMB) substrate (SeraCare Life Sciences, Gaithersburg, MD, United States) was added and the samples were incubated at room temperature in the dark for 15 min. The reaction was terminated by the addition of 2 M H_2_SO_4_. The optical density at 450 nm (OD_450_) was measured using an automatic ELISA plate reader (AccuReader, Metertech, Taipei, Taiwan R.O.C.). All samples and controls were run in duplicate.

To determine the IgG subclass responses (IgG1 and IgG2), dilutions of the specific antibodies were used as is shown in Table 2. The indirect ELISA protocol has been described above.

**Table 2:** Monoclonal and polyclonal antibodies (mAbs) used in this study for flow cytometry analysis and immunological assay.

**mAbs**	**Conjugated**	**Clone**	**Host**	**Dilution**	**Source**
Ant-CD3 epsilon	APC/Cy7	PC3/188A	Mouse	1:150	NBP2-54405APCCY7 (Novus, United States)
Anti-CD4	Alexa Fluor® 647	CC8	Mouse	1:100	MCA1653A647 (AbD Serotec, United Kingdom)
Anti-CD8	RPE	CC63	Mouse	1:100	MCA837PE (AbD Serotec, United Kingdoem)
Anti-IFN-gamma	Alexa Fluor® 405	345025	Rat	1:30	IC2300V-100UG (R&D Systems, United States)
Anti-TNF alpha	Alexa Fluor® 488	CC327	Mouse	1:200	MCA2334A488 (AbD Serotec, United Kingdom)
Anti-bovine IgG1	HRP	IL-A60	Mouse	1:2000	MA5-16736 (Invitrogen, United Kingdom)
Anti-bovine IgG2	HRP	IL-A73	Mouse	1:2000	MA5-16737 (Invitrogen, United Kingdom)
Anti-bovine IgG (H+L)	HRP	Polyclonal	Goat	1:2000	14-12-06 (KPL, United States)

*APC/Cy7, allophycocyanin—cychrome 7; RPE, R-phycoerithrin; HRP, horseradish peroxidase*.

### Peripheral Blood Mononuclear Cell (PBMC) Isolation and *in vitro* Culture

Peripheral blood mononuclear cell (PBMC) isolation was performed according to the method described in a previous study (39). Jugular or tail blood samples collected at week 10 of the experiment, that had been kept in ethylene diamine tetraacetic acid (EDTA) tubes (BD Vacutainer, Plymouth, United Kingdom), were diluted with sterilized PBS (pH 7.2; 1:1 dilution) and overlaid on 5 ml of Lymphoprep™ (STEMCELL Technologies, Vancouver, Canada) in 15 ml conical tubes. They were then centrifuged using a swinging bucket rotor at 400 × g at a temperature of 25°C and an acceleration of nine with no break for 30 min. The PBMC layer was carefully collected from the tube and transferred to a new 50 ml conical tube. The contaminated red blood cells were lysed by the 1 × red blood cell lysis buffer for 3 min at room temperature. PBMCs were then washed twice with PBS by centrifugation at 700 × g for 5 min at 25°C. The pellets were then resuspended with complete RPMI medium [RPMI 1640 medium supplemented with 1X antibiotic-antimycotic (100 units/ml of penicillin, 100 μg/ml of streptomycin, and 0.25 μg/ml of amphotericin B; Gibco™, Life Technologies Waltham, MA, United States) and 10% fetal calf serum (FCS, Gibco™)]. Cell viability was assessed by employing the Trypan Blue exclusion test carried out in a 0.1 mm Bürker chamber. Cells were then cultured in 24-well cell culture plates (2 × 10^6^ cells/well). PBMC cells were re-stimulated with rBbAMA-1(I/II) at a concentration of 30 μg/ml that had been obtained from the previous step. Concanavalin A (2.5 μg/ml, eBioscience, Carlsbad, CA, USA) and RPMI medium were used as positive and negative controls, respectively. Plates were incubated for 72 h at 37°C in an atmosphere containing 5% CO_2_.

### Cytokine Gene Expression Profiles Analyzed by Real-Time Quantitative PCR

To investigate the effects of rBbAMA-1(I/II) on cytokine expression, total RNA was extracted from cultured PBMCs, which were then stimulated with antigen using a PureLink™ RNA Mini Kit (Invitrogen, San Diego, CA, United States) according to the manufacturer's instructions. The concentration and quality of RNA were determined using a spectrophotometer. Total RNA (1 μg) was reverse-transcribed to synthesize cDNA using a Tetro™ cDNA Synthesis Kit (Bioline, London, United Kingdom) according to the manufacturer's instructions. The cDNA obtained from each sample was then used as a template to measure the expression of some genes involved in the inflammatory response. Real-Time quantitative PCR was carried out in the CFX96 Touch™Real-Time PCR (Bio-Rad, Hercules, CA, United States). A SensiFAST™ SYBR® Lo-ROX Kit (Bioline, London, United Kingdom) was used in an optimized 20 μl reaction volume according to the manufacturer's instructions. The reaction mixture contained 10 μl of 2x SensiFAST SYBR Lo-ROX Mix, 0.8 μl of 10 μM each primer, 2 μl of cDNA and 6.4 μl of RNase-free water. The real-time PCR conditions were as follows: an initial denaturation step at 95°C for 2 min followed by 40 cycles of 95°C for 10 s and an annealing step at 58°C for 30 s. The expression of each gene was normalized relative to the expression of Glyceraldehyde-3-phosphate dehydrogenase (*GAPDH*). Genes involved in the inflammatory response, including inducible nitric oxide synthase (*iNOS*), Interleukin *(IL)-2, IL-4, IL-6, IL-10*, interferon *(IFN)-*γ and tumor necrosis factor *(TNF)-*α were analyzed. Sequences of the primers used in this Real-Time PCR are presented in Table 3. The expression levels (fold-difference) of each gene were calculated using the 2^−ΔΔCT^ method (45).

**Table 3:** Primer sequences for SYBR green real-time quantitative PCR.

**Gene**	**Primer sequence (5'−3')**	**Annealing temperature (**°**C)**	**Product size (bp)**	**Reference**
*GAPDH*	F: GGCGTGAACCACGAGAAGTATAA	58	119	(40)
	R: CCCTCCACGATGCCAAAGT			
*iNOS*	F: AGCGGAGTGACTTTCCAAGA	58	97	(41)
	R: TTTTGGGGTTCATGATGGAT			
*TNF-α*	F: TCTTCTCAAGCCTCAAGTAACAAGT	58	103	(40)
	R: CCATGAGGGCATTGGCATAC			
*IFN-γ*	F: GATTCAAATTCCGGTGGATG	58	110	(42)
	R: TTCTCTTCCGCTTTCTGAGG			
*IL-2*	F: GGATTTACAGTTGCTTTTGGAGAAA	58	165	(43)
	R: GCACTTCCTCTAGAAGTTTGAGTTCTT			
*IL-4*	F: AGTGCTGGTCTGCTTACTGG	58	111	(42)
	R: TTCTTTCTCGTTGTGAGGATG			
*IL-6*	F: CACTCCAGAGAAAACCGAAGC	58	164	(40)
	R: GAAGCATCCCGTCCTTTTCCTC			
*IL-10*	F: CTTGTCGGAAATGATCCAGTTTT	58	84	(44)
	R: TTCACGTGCTCCTTGATGTCA			
*IL-12*	F: AACCTGCAACTGAGACCATT	58	186	(41)
	R: ATCCTTGTGGCATGTGACTT			

### Functional Characterization by Intracellular Cytokine Staining (ICS) and Flow Cytometry Analysis

For functional characterizations of the CD4^+^ and CD8^+^ cells, PBMC cells were stimulated *in vitro* as has been mentioned above. Brefeldin A (2 μg/ml; Sigma, St. Louis, MO, USA) was added for the last 6 h to facilitate intracellular cytokine accumulation. Following incubation, the PBMC cells were washed twice with staining buffer (1X PBS, 0.01% NaN_3_, 10% FBS) and were subsequently incubated with the respective anti-bovine monoclonal antibodies (CD3- APC/Cy7, CD4- Alexa Fluor® 647, CD8-RPE; Table 2). Cells were then fixed and permeabilised with Intracellular Fixation & Permeabilization Buffer Set (eBioscience, Carlsbad, CA, USA) and stained intracellularly with anti-bovine IFN-γ-Alexa Fluor® 405 and TNFα- Alexa Fluor® 488 antibodies (Table 2). Flow cytometry was performed by acquiring 10,000 events in the live lymphocyte gate using DxFLEX Flow Cytometer (Beckman Coulter, Brea, CA, United States) and further analyzed using CytExpert for DxFLEX Software.

### Statistical Analysis

Statistical analysis was carried out using Graph Pad Prism 8.0.2 (GraphPad Software, Inc., San Diego, CA, United States). Statistical significance between groups was determined by one-way ANOVA or non-parametric ANOVA. Values of *P* < 0.05 were taken to be statistically significant. The results of the assumption test were analyzed before statistical analysis was performed.

## Results

### Production of the rBbAMA-1(I/II) Protein

**Figure 1:**
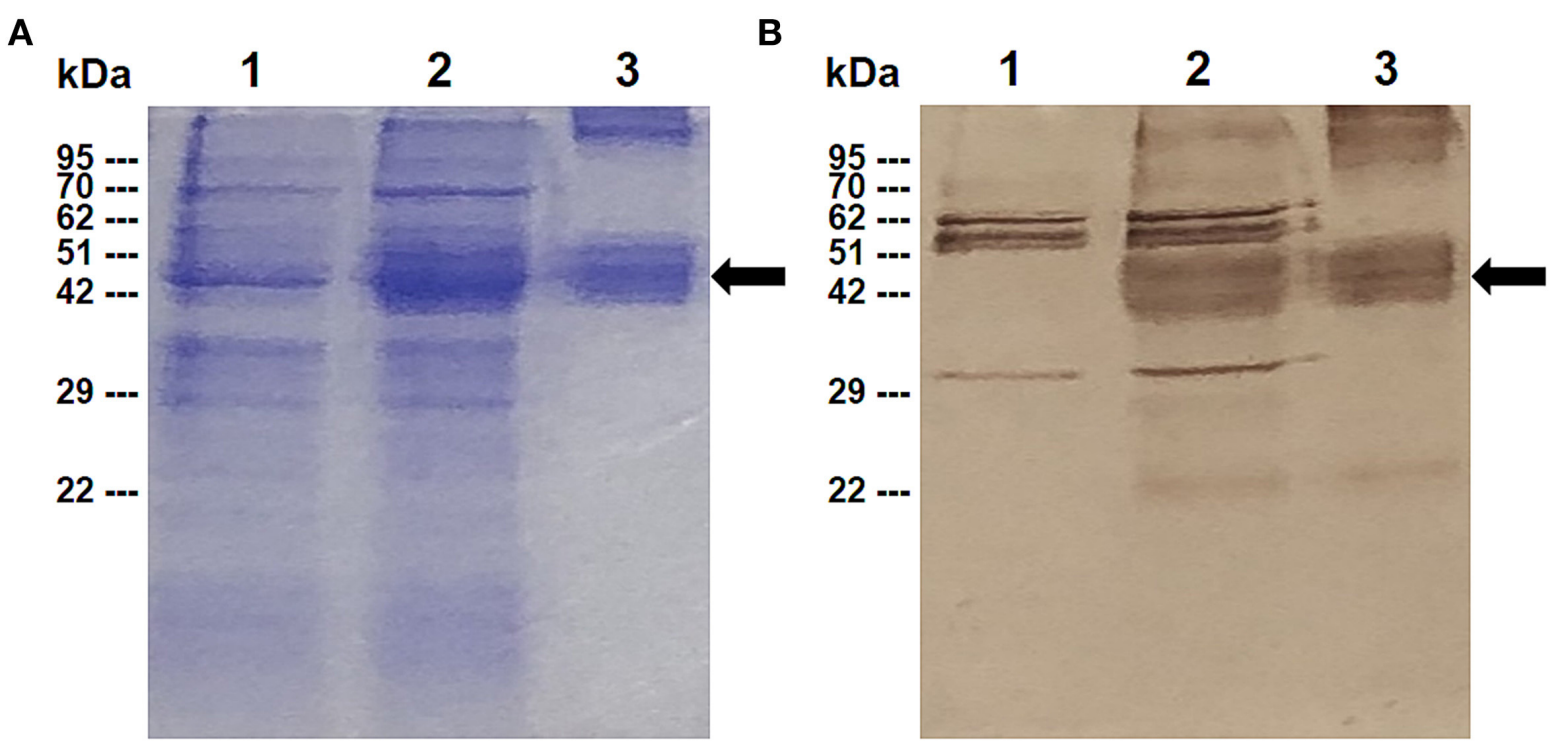
Expression of rBbAMA-1(I/II) analyzed using SDS-PAGE stained with Coomassie brilliant blue **(A)** and western blotting analysis **(B)**. Lane 1: Non-induced *E. coli* strain M15 containing pQE-32/BbAMA-1(I/II) plasmid; Lane 2: Induced *E. coli* strain M15 containing pQE-32/BbAMA-1(I/II) plasmid; Lane 3: Purified rBbAMA-1(I/II) protein. Black arrows indicate the target protein band of rBAMA-1(I/II).

The expression of the rBbAMA-1(I/II) protein was analyzed by SDS-PAGE (Figure 1A) and western blotting analysis (Figure 1B). The overexpression of the target protein band was observed to be ~48 kDa on SDS-PAGE gel. Western blotting analysis confirmed that the rBbAMA-1(I/II) protein was successfully expressed in this study, as was established through the use of the specific-sBbAMA-1 antibody.

### Humoral Immune Response Induced by rBbAMA-1(I/II)

**Figure 2:**
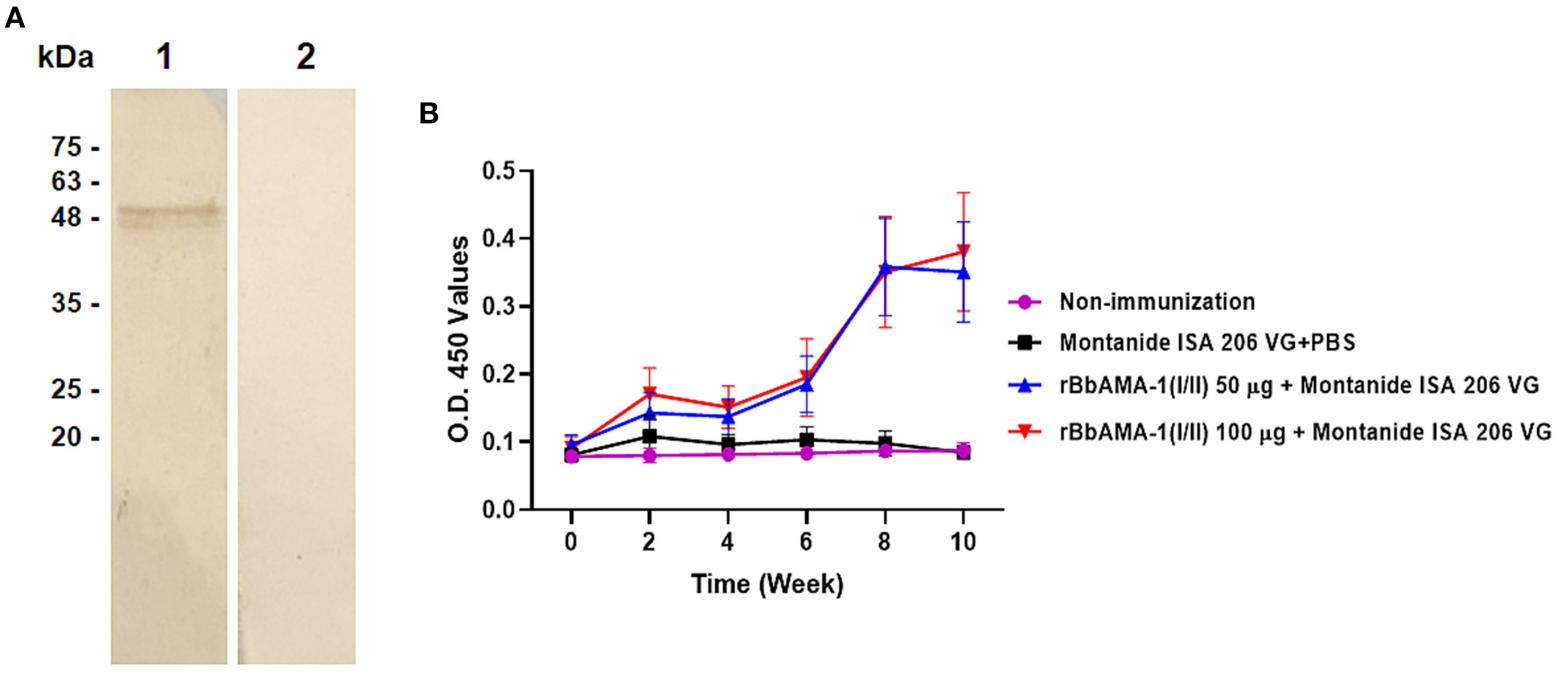
Determination of rBbAMA-1(I/II)-specific antibody response. **(A)** Western blotting analysis demonstrated that pooled cattle serum immunized with rBbAMA-1(I/II) reacted with the purified recombinant protein (lane 1) when compared to the pre-immunization period (lane 2). **(B)** Indirect ELISA test revealed that the antibody response continuously increased after the first immunization with rBbAMA-1(I/II).

In order to characterize the antibody response elicited by rBbAMA-1(I/II), the cattle sera that were immunized with rBbAMA-1(I/II) were evaluated using western blotting analysis and indirect ELISA. The results revealed that the pooled sera of the rBbAMA-1(I/II)-immunized groups collected from week 10 specifically reacted to the purified rBbAMA-1(I/II) at ~48 kDa on the nitrocellulose membrane (Figure 2A).In consideration of the antibody response, the specific IgG to purified rBbAMA-1(I/II) was detected among the rBbAMA-1(I/II)-immunized groups. Remarkably, the antibody titers continuously increased throughout the experiment when compared to those of the control groups (Figure 2B).

**Figure 3:**
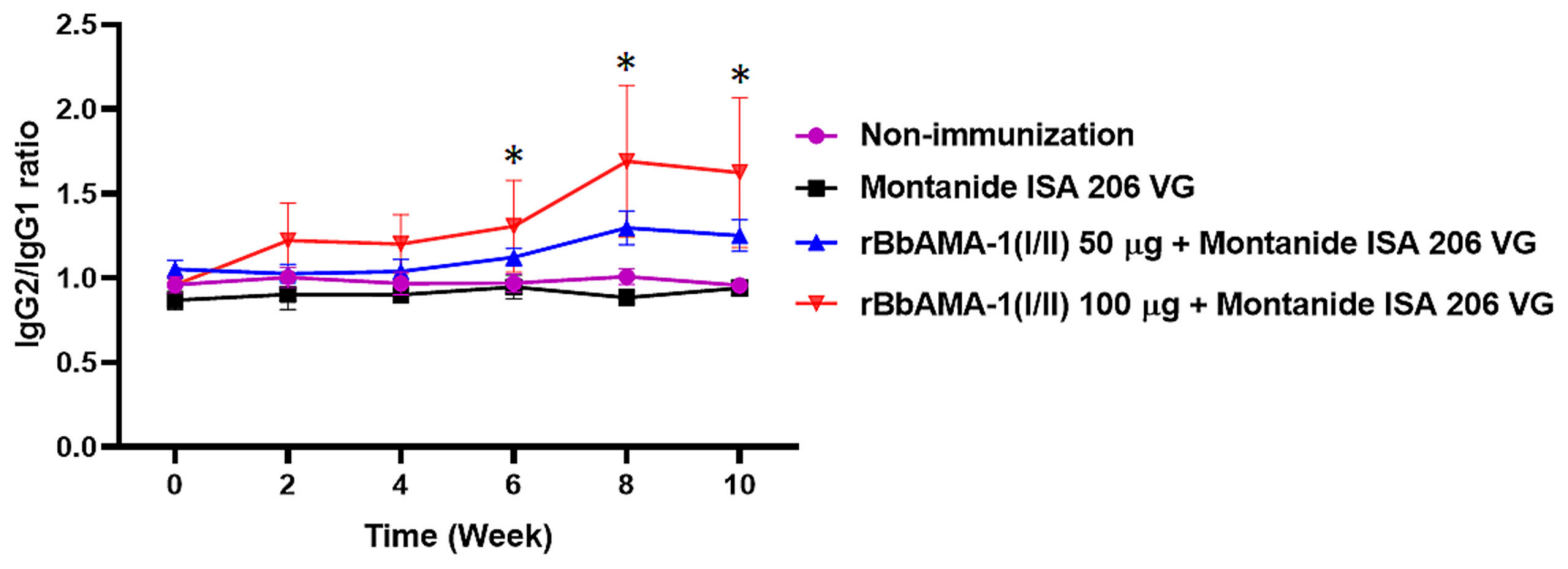
Time course of IgG2/IgG1 ratio response to rBbAMA-1(I/II) assessed by indirect ELISA. Each time point depicts the group mean value ± SD of the IgG2/IgG1 ratio. Asterisks indicate a significant difference (*P* < 0.05).

Furthermore, an indication of the bias toward the T helper type 1 (Th1) cell response was also characterized by measuring the IgG1 and IgG2 bovine isotypes in the present study. The IgG2/IgG1 ratio >1 was an indicator of a Th1 type response. As a result, the ratio of IgG2/IgG1 in cattle immunized with 100 μg of the rBbAMA-1(I/II) group was higher than for the cattle immunized with 50 μg of rBbAMA-1(I/II) and the control groups. As observed, this ratio increased in the vaccinated groups throughout the course of the experiment. However, there was no statistical significance in the IgG2/IgG1 ratio among the rBbAMA-1(I/II)-immunized groups (Figure 3). Additionally, statistically significant differences were observed over the last 3 weeks, especially in cattle immunized with 100 μg of the rBbAMA-1(I/II) when compared with those in the control groups.

### The rBbAMA-1(I/II) Induced Cellular Immune Responses

**Figure 4:**
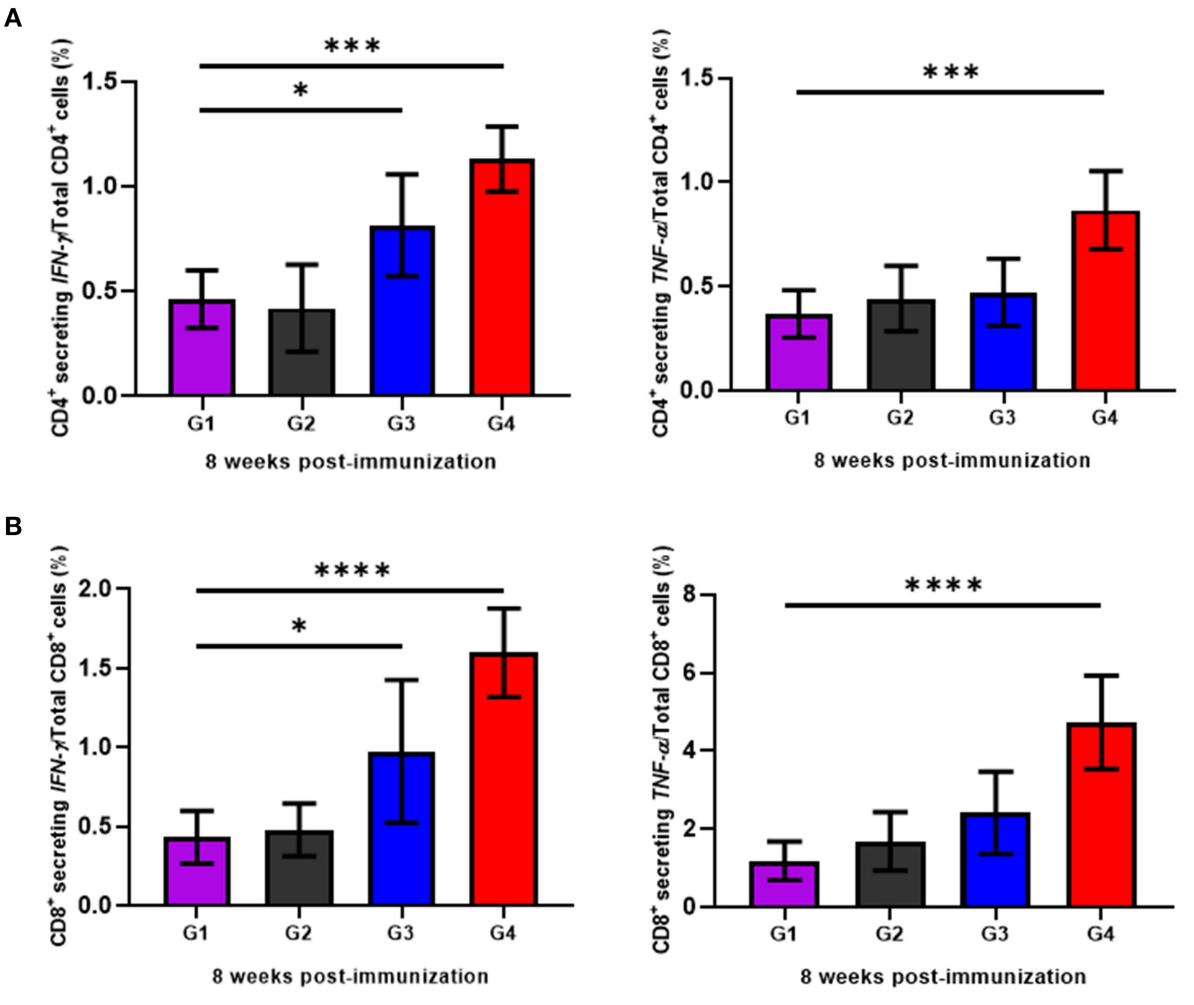
Determination of cellular immune response of cattle immunized with rBbAMA-1(I/II) analyzed by flow cytometry analysis. **(A)** Frequency of IFN-γ and TNF-α secreting CD4^+^ T cells. **(B)** Frequency of IFN-γ and TNF-α secreting CD8^+^ T cells. The results are expressed as group mean values ± SD. Asterisks indicate a significant difference (* < 0.05; *** < 0.001; **** < 0.0001). Experimental groups G1 = non-immunization, G2 = Montanide ISA 206 VG + PBS, G3 = rBbAMA-1(I/II) 50 μg + Montanide ISA 206 VG and G4 = rBbAMA-1(I/II) 100 μg + Montanide ISA 206 VG.

To determine the cellular immune responses induced by rBbAMA-1(I/II), peripheral blood mononuclear cells (PBMCs) isolated from animals in each group were cultured *in vitro* and re-stimulated with rBbAMA-1(I/II). Subsequently, flow cytometry analysis was performed to characterize the CD4^+^ and CD8^+^ T cell response by producing pro-inflammatory cytokines (IFN-γ and TNF-α). The present findings demonstrate that rBbAMA-1(I/II) could trigger bovine PBMCs to elevate the proportion of antigen-specific CD4^+^ T cell secreting IFN-γ with statistical significance in both groups of cattle immunized with 50 μg (*P* < 0.05) and 100 μg of rBbAMA-1(I/II; *P* < 0.01), as is shown in Figure 4A. Furthermore, 100 μg of rBbAMA-1(I/II) significantly produced higher percentages of CD4^+^ T cell secreting TNF-α (*P* < 0.01) in comparison with the control groups. With regard to the CD8^+^ T cells, the present study found that both rBbAMA-1(I/II) immunized groups elicited high frequencies of CD8^+^ T cell secreting IFN-γ with statistical significance (*P* < 0.05 and *P* < 0.0001 for 50 and 100 μg of the rBbAMA-1(I/II), respectively). Meanwhile, high proportions of antigen-specific CD8^+^ T cell secreting TNF-α were detected in cattle immunized with 100 μg of the rBbAMA-1(I/II) with statistical significance (*P* < 0.0001) (Figure 4B).

**Figure 5:**
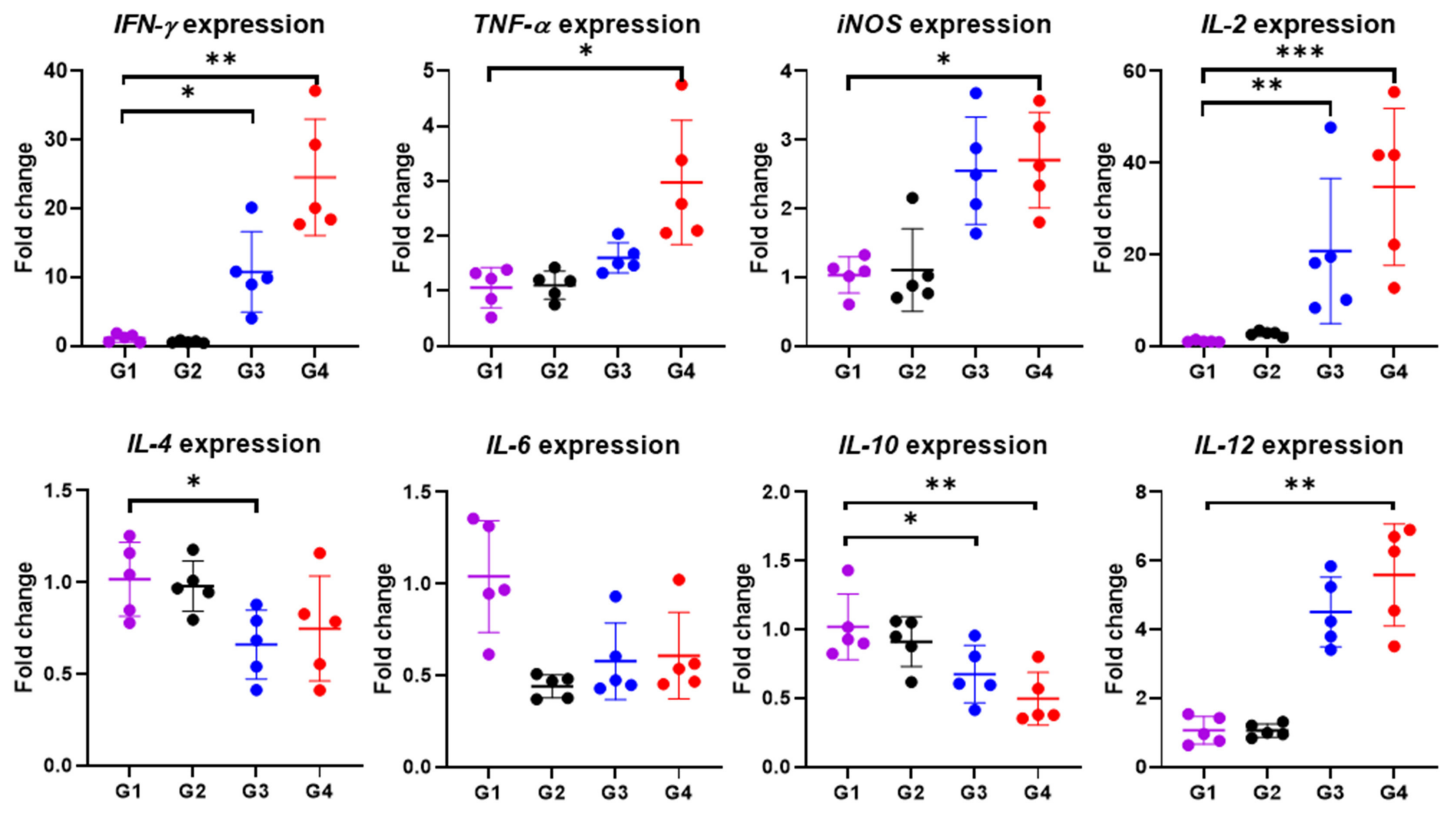
Inflammatory cytokine gene expression profiles induced by rBbAMA-1(I/II). The results are expressed as mean values ± SD. Asterisks indicate a significant difference (* < 0.05; ** < 0.01; *** < 0.001). Experimental groups G1 = non-immunization, G2 = Montanide ISA 206 VG + PBS, G3 = rBbAMA-1(I/II) 50 μg + Montanide ISA 206 VG and G4 = rBbAMA-1(I/II) 100 μg + Montanide ISA 206 VG.

In particular, cytokine gene expression levels were also investigated in order to gain an insight into cellular response at the gene level. A significant up-regulation among cytokine genes, including *IFN-*γ (*P* < 0.05), *TNF-*α (*P* < 0.01), *IL-2* (*P* < 0.001), *IL-12* (*P* < 0.01) and *iNOS* (*P* < 0.05) in cattle immunized with 100 μg of the rBbAMA-1(I/II), were observed after the rBbAMA-1(I/II)-stimulated cultures were compared with the control groups (Figure 5). Higher expression levels of pro-inflammatory cytokines *IFN-*γ and *IL-12* were observed. Although cattle immunized with 50 μg of rBbAMA-1(I/II) induced cytokine gene expression, no significant differences were observed with the exception of *IL-2* (*P* < 0.01) and *IFN-*γ (*P* < 0.05). In contrast, mRNA transcription of *IL-4, IL-6* and *IL-10* were down-regulated when compared with the control groups. In addition, *IL-10* gene expression was significantly down-regulated in cattle immunized with 100 μg of the rBbAMA-1(I/II) group (*P* < 0.01).

## Discussion

*Babesia bovis* is an economically important pathogen known to cause bovine babesiosis worldwide. Due to the fact that the vaccines currently being administered are neither fully safe nor effective, researchers have extensively searched for a candidate vaccine antigen that could be used to develop an effective vaccine against *B. bovis* (4). As has been found with BbAMA-1, studies of its immunogenicity demonstrated its vaccine potential (12, 36, 37). However, a lack of in-depth knowledge and understanding of the immunological characterization of the response may have implications for the design and assessment of AMA-1-based vaccines for bovine babesiosis. Therefore, basic research efforts involving the identification and characterization of the immunological function would be very important in the development of recombinant vaccines. In the present study, the ectodomains I and II region of BbAMA-1 has been successfully expressed as a doublet band corresponding to around 48 kDa. Because BbAMA-1 encompasses the extracellular cysteine-rich domains I and II (12, 36, 37). It is likely that cysteine oxidation and formation of intra-molecular disulfide bonds is responsible for the doublet formation in Laemmli SDS-PAGE. Moreover, doublet in SDS-PAGE may result from proteolytic degradation (46, 47). However, the doublet bands were identical as the same protein identified by anti-sBbAMA-1 serum, which is specific to 50 amino acid residues of recombinant BbAMA-1(I/II) protein. Therefore, it was used for further immunization.

Determining the functional regions of the *B. bovis* antigens could be one of the best strategies for blocking this parasite's biological functions during host cell entry (48). It has been suggested that AMA-1 participates in the invasion stage, which is conserved for most apicomplexa. Immunological investigations in the present study showed that rBbAMA-1(I/II) could induce a humoral immune response, for which the IgG2 isotype response was predominantly higher than IgG1 isotype. Enhancement of IgG2 over the IgG1 antibody response indicates a bias toward the Th1 cell response (49). The evidence of IFN-γ detected in our present study is one of the justifications for the predominant IgG2 isotype (50). Generally, it has been suggested that the bovine IgG2 isotype possesses more functional opsonic characteristics than IgG1 in enhancing phagocytic activity and NO production (51). Interestingly, it has been reported that a higher IgG2a/IgG1 ratio is associated with a protective immune response against the intracellular pathogen (52). Therefore, evidence of the fact that IgG2 response in this study was higher than IgG1 indicated the possibility that rBbAMA-1(I/II) was capable of providing protective immunity against intracellular protozoan *B. bovis* infection.

To defeat the *Babesia* pathogen, not only is a humoral immune response important in eliminating the parasite, but so is a cellular immune response (1). Therefore, a cellular immune response has been studied and described in the present study in an attempt to gain a more comprehensive understanding of rBbAMA-1(I/II) immunological characteristics so as to evaluate its vaccine performance. In *Plasmodium*, CD4^+^ T cells displayed a central role against parasitized erythrocytes after being activated by antigen-presenting cells during blood-stage malaria (53). Interestingly, IFN-γ and TNF-α producing CD4^+^ T cells play a crucial role in the protective immune response to the blood-stages of the malaria parasite in humans (54, 55). Remarkably, the secretion of both IFN-γ and TNF-α from the same T cell would be more effective than cytokine alone for killing (56). Previous studies have also suggested that CD4^+^ T cells and IFN-γ are required to establish protective immunity against infection with *Babesia* parasites (57, 58). It has been determined that IFN-γ is involved in the protective innate immune response to *B. bovis* in calves (59–61). Therefore, it has been proposed that vaccines that prime CD4^+^ T cells to produce IFN-γ could induce and provide protective immunity against *Babesia* infection (4). Previously, the search for *B. bovis* candidate antigens as subunit vaccines has determined that some antigens have the potential to elicit CD4^+^ T cells in producing IFN-γ or TNF-α such as Bb-1 (62), Bbo20 (63), rhoptry-associated protein 1 (RAP-1) (64), small heat shock protein (Hsp20) (65) and merozoite surface antigens (MSAs) (66). Likewise, the flow cytometry analysis conducted in our study found that CD4^+^ secreting IFN-γ/TNF-α T cells was detected in cattle that had been immunized with rBbAMA-1(I/II). In support of this result, the up-regulation of IFN-γ and TNF-α mRNA transcription was noted. In this regard, the presence of IFN-γ cytokine indicates that the Th1 cell response is required to mediate protection against a variety of intracellular infections (67). In accordance with the antibody response obtained from our study, we have verified the immunological role of BbAMA-1(I/II) in induction of Th1 response against *B. bovis*. Therefore, it can be concluded that the rBbAMA-1(I/II)-based vaccine formulated in this study may provide effective protective antibodies against *B. bovis* infection.

Apart from the CD4^+^ T cell response, CD8^+^ T cells are vital in offering protection against liver-stage malaria (53). Unlike *Plasmodium* parasites, the pre-erythrocytic stage is not found in the *Babesia* parasite (68). Therefore, the majority of the immune responses against *Babesia* is dependent upon the CD4^+^ T cell response rather than the CD8^+^ T cell response (4). However, CD8^+^ T cells still play an important role in contributing to the clearance and immune memory against many intracellular pathogens, as has been observed in many incidences of malaria parasite infection (53). Currently, a number of studies have reported that CD8^+^ T cells are associated with the control of the *Babesia* parasite (60, 69). Interestingly, high percentages of antigen-specific CD8^+^ secreting IFN-γ/TNF-α T cells have been detected in our study. The CD8^+^ T cells secreting IFN-γ/TNF-α may have enhanced cytolytic activity (56). In this case, the generation of robust CD8^+^ responses would likely be relevant in vaccinations. This would likely be due to the fact that Th1 CD4^+^ T cells provide assistance in establishing optimal CD8^+^ T cell effector activity (70). Therefore, if sufficient antigen-specific CD8^+^ T cell responses are generated and provided against the vaccine, disease can be prevented and controlled.

In addition to T cell lymphocytes, macrophages are the key effector cells that mediate the clearance of pathogens by killing the organisms associated with phagocytosis in innate immune response and by regulating the consequent adaptive immune responses (4, 68). Previous studies have demonstrated that macrophages are critical for establishing protective immunity and resistance to *Babesia* infection in mice (71). Furthermore, they are responsible for producing an inflammatory cytokine of nitric oxide (NO) *via* the co-stimulation of biologically active IFN-γ and TNF-α or the presence of parasite derived products (72). Interestingly, it has been determined that NO has been shown to exhibit babesiacidal activity (73). Therefore, the resistance to babesiosis appears to correlate with an increase in NO production (58). Although, active NO levels were not determined in our study, the *in vitro* up-regulation of mRNA expression by *iNOS*, a key enzyme in the macrophage inflammatory response, was observed among cattle that had been immunized with rBbAMA-1(I/II) groups when compared with the control groups. This observation is likely related to the presence of the CD4^+^ secreting IFN-γ/TNF-α T cells or the indication that rBbAMA-1(I/II) may contribute to the enhancement of the transcription of *iNOS*. Consequently, this would imply that active NO production could possibly be induced by the rBbAMA-1(I/II)-based vaccine.

In addition to NO, other pro-inflammatory cytokines are involved in the defense against intracellular pathogens and are important in activating an innate and acquired immune response. Using real-time PCR, our results showed that not only were *IFN-*γ and *TNF-*α responsible for the Th1 response when up-regulated in cattle receiving rBbAMA-1(I/II), but that *IL-2* and *IL-12* were also directly involved. Interestingly, our results were similar to those of the study involving the AMA-1 of *Plasmodium yoelii* (*P. yoelii*), for which significantly higher levels of bioactive IFN-γ, TNF-α and IL-2 were noted in the immunized group (22). Notably, previous studies have reported that the multifunctional Th1 cells simultaneously expressing IFN-γ, TNF-α and IL-2 correlated best with the degree of protection of vaccine models against parasite infection (55, 74). These cells were classified as effector memory cells (74). With regard to IL-2, it was found to exhibit little direct effector function; however, it appears to be a promoter of CD4^+^ and CD8^+^ T cells expansion in serving the effector T-cell responses. Furthermore, it could enhance the memory capacity and effector function of CD8^+^ T cells and natural killer (NK)-cell activity. Therefore, cytokine markers of IL-2, TNF-α and IFN-γ can provide a relatively simple set of cytokines that could be used to define a vaccine-elicited response against specific infections that require T cells for protection (56). During the acute stage of babesiosis, the resistance is associated with early transcriptional up-regulation of *IL-12* and *IFN-*γ (59, 75). IL-12 is a Th1 cytokine produced by activated monocytes and macrophages. Bioactive IL-12 develops and maintains the Th1 cells by activating Th1 cells and NK cells to produce IFN-γ (76, 77). Importantly, IL-12 plays an important role in the inhibition of Th2 differentiation (76). Therefore, the significant down-regulation of *IL-4* and *IL-10* transcription among cattle immunized with rBbAMA-1(I/II) in our study was likely due to IL-12 cytokine suppression.

Given that the structural BbAMA-1 domains I and II could elicit both humoral and cellular immune responses along with predominantly Th1 responses, it is interesting to employ this antigen as a vaccine candidate against bovine babesiosis. Regarding strain variations that dramatically interfere with the live vaccine efficacy and are an obstacle in the development of recombinant vaccines, BbAMA-1 which is highly conserved and distributed throughout *B. bovis* would help to address issues of ethnicity by providing protective immunity across *B. bovis* strains in the field condition. To complete the evaluation of its vaccine potential, this vaccine formulation will be used in future work in order to determine protection efficacy against challenge infection with a virulent strain. The findings reported here should be useful in better understanding and improving the design of BbAMA-1-based vaccines against *B. bovis* infections.

## Data Availability Statement

The datasets presented in this study can be found in online repositories. The names of the repository/repositories and accession number(s) can be found below: https://www.ncbi.nlm.nih.gov/genbank/, KY575957.

## Ethics Statement

The animal study was reviewed and approved by the Faculty of Veterinary Medicine, Chiang Mai University Animal Care and Use Committee (FVM – ACUC). Written informed consent was obtained from the owners for the participation of their animals in this study.

## Author Contributions

AR, VP, ST, and NS conceived and designed the experiments and performed the data analysis. AR, BN, AM, NA, PK, WS, PC, and KS collected samples and performed the experiments. AR and NS wrote the manuscript. AR, VP, ST, NY, and NS directed the analyses and revised the manuscript. All authors contributed to the article and approved the submitted version.

## Funding

This work was supported by the Royal Golden Jubilee Ph.D. Programme Scholarship, Grant No. PHD/0224/2560. This work was also supported by the National Research Council of Thailand, Grant No. FF65/089. The funders had no role in study design, data collection and analysis, decision to publish, or preparation of the manuscript.

## Conflict of Interest

The authors declare that the research was conducted in the absence of any commercial or financial relationships that could be construed as a potential conflict of interest.

## Publisher's Note

All claims expressed in this article are solely those of the authors and do not necessarily represent those of their affiliated organizations, or those of the publisher, the editors and the reviewers. Any product that may be evaluated in this article, or claim that may be made by its manufacturer, is not guaranteed or endorsed by the publisher.
